# Mr. J. H. James on the Principles, Nature, and Treatment of the Different Species of Inflammation

**Published:** 1822-03-01

**Authors:** 


*Observations on some of the general Principles and on the particular Nature and Treatment of the different Species of Inflammation; being, with Additions, the Substance of an Essay to which the Jacksonian Prize for* 1818 *was adjudged, by the Royal College of Surgeons.* By J. H. James, Surgeon to the Devon and Exeter Hospital, and Consulting Surgeon to the Exeter Dispensary. London, 1821. Octavo, pp. 305.

---

"The knowledge of inflammation, in all its variety of causes, effects, terminations, and method of treatment, may be truly said to constitute the basis of scientific surgery, entering, more or less, into the prevention or cure of every disease which comes under the surgeon's care." Mr. Wilson's Lectures, p. 243.

---

The respect which we entertain for the opinions of the distinguished individuals who preside over the London College of Surgeons, naturally induces us to approach a work which has been honoured by their favourable suffrage, with more confident hopes of finding important information, than if it did not possess such an honourable passport. Let it not be imagined, however, that any favourable prepossessions of the merits of the work before us, even founded upon such a basis as the approbation of the College of Surgeons, will induce us to forego our duty as impartial reviewers. We shall comment upon its contents with our usual sincerity, from a perfect accordance with a quotation supplied by our author himself, that, "nothing has been so great an obstacle to the improvement of science, as the partiality or obsequious regard which men have been too apt to pay to great authorities."

The vast importance of the subject of inflammation must be so evident to every physician and surgeon, and it has been so frequently insisted upon, that it would be a waste of time to obtrude any arguments for the purpose of demonstrating that the practitioner, who is not intimately acquainted with every part of it, can neither execute his professional duties with satisfaction to himself, nor advantage to his patient.

Mr. James justly observes, that although Mr. Hunter's work on inflammation has laid an ample and durable foundation for such an edifice of knowledge as may fulfil all the purposes of science in this department; yet, to the superstructure much remains to be added, before it can be considered perfect and complete. The present Essay, however, is not submitted to the profession as a systematic work; it is avowedly much too concise upon many points, to warrant the assumption of such a title.

The inquiry into the subject of inflammation divides itself, according to our author, into various branches. 1st. The state of the vessels and nerves, in the part inflamed, constituting the immediate cause of the phenomena we observe. 2dly. The general laws of the economy, and chiefly the phenomena of sympathy, as they relate to disease in general, and to inflammation in particular; as also the circumstances belonging to, or affecting the individual, which are capable of influencing its progress and results. 3dly. The objects of inflammation in the animal economy, the phenomena which it exhibits, the effects which it produces, and the products which it affords. 4thly. The general principles of treatment, which have been so ably considered by various modern authors, that in the present work this part of the subject is wholly avoided. 5thly. In the words of our author, "a particular arrangement of the different kinds of inflammation, I am justified in saying, is very much wanted, and it has been a principal object of my endeavours in this essay to effect this." It is not contended that there are not arrangements of inflammation in existence, proposed even by authors of celebrity; that those, however, which preceded Mr. Hunter were to him unsatisfactory, may be concluded from his having omitted all mention of them. From the opinion of an author of late date and acknowledged authority also, (Dr. Thompson,) it appears that none have subsequently been offered, which is entitled to command our attention. 6th and lastly. To consider the peculiarities of each inflammation, and to indicate the principles of treatment particularly adapted to it. "In all modern works," says Mr. James, "and for the most part in those of more ancient writers, the views of inflammation which have been given are, as it appears to me, too general; and this observation also extends to treatment, which has commonly been laid down under certain heads; such as the antiphlogistic plan—bleeding, cold, &c. It seems, however, important to specify exactly the case in which one mode or one remedy is more likely to succeed than another."

From these observations it would appear to us, that our author had consulted *only* those general views, which, in systematic works, properly precede the consideration of the nature of, and the peculiar treatment demanded in, the different species of inflammation. We really are not aware that this important part of the subject has escaped the attention of previous writers.

Mr. James has divided his work into two parts; the first containing "Observations on some of the General Principles of Inflammation;" the second being occupied by a description Of the different species of Inflammation."

Having thus given an outline of the purposes which it is the author's intention to fulfil, we shall proceed to submit to our readers an abstract of the most prominent parts of his work.

Chap. 1st. "On the Principles on which an Arrangement of Inflammations may be founded." A cursory statement of the modes of dividing or arranging the subject now in use, precedes the exposition of the principles upon which Mr. James has founded his arrangement.

1st. Inflammations are commonly treated of as acute, subacute, or chronic, but these it is observed are merely different stages of the same state in many instances. 2dly. As the adhesive, suppurative, ulcerative, or gangrenous "processes, which cannot be too attentively considered; but they are merely modes of termination, often of different kinds, but sometimes of the same; to a certain extent they will afford a basis of distinction, but to me it seems that this is only in as far as they constitute a ground work for *subdivisions*, as will be hereafter explained." 3dly. As phlegmonous, erysipelatous, or gangrenous. The following objections to this division of inflammation are submitted:—

"Under these titles, diseases of the most opposite nature, and requiring the most opposite treatment, have been indiscriminately brought together. And supposing no objections occurred to the titles, (a point which will be hereafter considered,) would a division into three kinds be sufficient? Is it not looking at this numerous class of diseases in too narrow a point of view? Are we to treat them upon principles derived from such a limited consideration? Is not this contenting ourselves with ideas at once too general and too vague?

"A separate division under the title of gangrenous inflammations is hardly to be admitted. For how are we to limit them? Many of the diseases which would be included under the head of phlegmonous and of erysipelatous inflammations, terminate in gangrene; some inevitably, others only incidentally. Are they all to be called gangrenous? Are the classes of phlegmonous and erysipelatous inflammations to be robbed of them at one time and not at another? or how are they to be definitively arranged? The disposition to terminate in gangrene will afford a basis for subdivision, but not for a primary separation." 5.

We are indebted to Dr. Carmichael Smith in this country, and to Pinel and Bichat on the Continent, for another principle of arrangement, which is grounded upon the elementary tissues, in which inflammations occur. These tissues are five in number, and this doctrine supposes, that the inflammation of each is essentially different; that of the first, which is the cellular membrane, including the parenchyma of the various viscera, is considered *phlegmonous;* of the second, *serous*, from the membranes in which it is seated; of the third, *mucous*, it is presumed, for the same reason; of the fourth, which is the skin, *erysipelatous;* and of the fifth, *rheumatic*, belonging to the fibrous membranes.

It cannot be denied, that the nature of inflammation differs materially, when it attacks different elementary tissues; but, when the doctrine is carried to the extent proposed by the abovementioned writers, it is, in the opinion of our author, and with which we fully concur, demonstrably erroneous. We shall give his arguments in his own words.

"This system" that of C. Smith, Pinel, and Bichat, "is liable to the following objections, fatal if true; namely,

"1*st.* That different kinds of inflammation are liable to occur in the same tissue. 2*d.* That the same kind of inflammation is often met with in different tissues. 3*d.* That the same inflammation shall be transferred from one to another: a position, I must grant, less susceptible of direct proof than the two former; but either are sufficient for my purpose.

"1*st.* That different kinds of inflammation are liable to occur in the same tissue.

"In the Skin there are erysipelas, the various exanthemata, and other cutaneous diseases, particularly ecthyma, which approaches to boil: we have those tumors still more analogous, called pinswells, and various other forms, as from blisters, scalds, poisonous substances, chilblains, mechanical injury, *&c.* which are not erysipelas, and which are not identified, because we choose to call them erysipelatous.

"2*d*. In Cellular Membrane. Besides true phlegmon, we meet with boil, carbuncle, erysipelas, scrofulous abscesses, *&c.* affections differing widely from each other.

"3*d*. Mucous Membranes, it is admitted, ulcerate in one part, effuse lymph in another, and secrete pus in a third; they are the subjects of catarrh, dysentery, angina, croup, of *various forms* of inflammation of the bronchia and intestinal canal, described by Badham, Hastings, Abercrombie, and others.

"4*th*. Serous Membranes are liable to inflammation of sthenic and asthenic type, if not to rheumatism; it is not always the *same*. There is, I conceive, no distinct *serous* inflammation, as has been maintained.

"5*th*. In Fibrous Structure we have rheumatism, gout, syphilis.

"Enough, I think, has been stated: the question lies in a narrow compass;—the tissues are five; are there more than five species of inflammation or not? If there are, one tissue must be liable to more than one, and of course there is an end of the doctrine. As far as it properly goes, the theory is valuable, but it has, I conceive, been pushed beyond proper limits." 7.

"2*dly*. That the same kind of inflammation is often met with in different tissues; the skin, mucous membranes, glands, periosteum, bone, *&c.* are liable to inflammation from syphilis; almost every structure in the body from scrofula; the urethra and bladder, if not the bowels, are sometimes affected by rheumatism. The inflammation in the diathesis of measles, small-pox, erysipelas, *&c.* may fix upon serous or mucous membranes, as well as skin, and often, if not generally, does so.

"3*d*. They are often transferred from one tissue to another.

"It is maintained, that when this happens, the new inflammation is different from the other. Upon this head we may observe, that proof cannot be obtained, because the organ so affected is generally internal; or if not, its structure is so different, that inevitably the form must vary. Thus, if inflammation is tranferred from the urethra to the testis, it is not gonorrhœa there; nor, if the reverse happens, is it hernia humoralis;—so, if erysipelas receding affects the brain, it may not actually be erysipelas, whose characters have been defined from its appearances in a different organ. But the inflammation, I conceive, is as analogous in its nature to it, as circumstances will permit, and differs in its nature from inflammation of the *same* organ arising from other causes. If inflammation recedes from an external organ, and attacks one internal, there doubtless will be a material change in the *constitutional sympathies*, and a proportional alteration in the *treatment* will be required; but *this* may be explained from the greater importance of the part. If the doctrine alluded to were true, then it would be sufficient, that any given tissue were inflamed, for its nature to be the same: a position so irreconcileable with facts, that its advocates admit that the cause may modify it; if it does so, this modification may be so material, as to throw the influence of the texture into the back ground,—and for this I contend." 9.

The objections maintained by our author, against this doctrine, are supported by the opinion of Mr. Hunter, who says, (vol. I. p 473)

" It has been supposed that, the different species or varieties of inflammation arise from the difference in the nature of the part inflamed, but this is certainly not the case, for if it was, we should soon be made acquainted with all the different inflammations in the same person, at the same time, and, even in the same wound. For instance, in an amputation of a leg, where we cut through the skin, cellular membrane, muscle, tendon, periosteum, bone, and marrow, the skin should give us the inflammation of its kind, the cellular membrane, of its kind, the muscles of theirs, the periosteum, bone, marrow, &c. of theirs; but we find, it is the same inflammation in them all."*

After taking a rapid survey of Mr. Hunter's divisions of, and observations upon, inflammation, which, we trust, are too deeply impressed upon the minds of our readers to require any particular notice from us, Mr. James proceeds to an explanation of the principles upon which the arrangement he proposes, is founded. Upon this part of the subject, our author may claim the merit of originality, at least, and we think, there are few who will not agree with us, that he has evinced also much ingenuity in his mode of treating it. We shall submit to our readers a copious extract from this part of the work.

" The arrangement of inflammations here proposed is founded upon the following facts:

" 1*st.* That the mode of repairing injury, and of arresting the progress of inflammatory diseases, depends upon the power which the animal œconomy possesses of effusing organizable lymph. If this exists in any given case, in a sufficient degree, the progress of the inflammation will be *limited;* if the contrary, it will *spread.*

" And the danger of the disease being in proportion to the disposition to spread *cæteris paribus*, more constitutional sympathy, denominated sympathetic fever, will be excited.

" And this sympathetic fever, however salutary in its nature, will, when it exceeds certain bounds, tend to augment rather than lessen the mischief.

" The disposition to spread may be owing either to the nature of the part, as a surface; of the cause, as a poison; or to the state of the constitution;—but the former are circumstances more or less *accidental*, and though very important, cannot afford a basis of dis-

* Mr. James quotes Mr. Hunter "from the edition in 8vo. by Mr. Travers." We find that, it is the last 8vo. edition, published by Cox, to which he refers. Mr. Travers has not, we believe, edited any of Mr. Hunter's works.

tinction: but the latter, as a *permanent* cause, certainly will, and when it is similar in nature and degree, and accompanied with the same concomitants, it will be found to produce the same effects; and there is no inflammation in which the disposition of the constitution does not tend either to produce its limitation or the reverse. These concomitants are very various, as we shall hereafter have occasion to specify.

"The disposition then to *limit*, or to *spread*, will afford the ground-work, by constituting two great classes of inflammation. It may be said, perhaps, that it would be as well to designate them by the old titles sthenic or asthenic, or by the terms phlegmonous and erysipelatous. It is not, however, difficult to shew that either of these are (is) liable to strong objections.

"The terms sthenic and asthenic might probably be employed with reference to internal inflammations without any obvious disadvantage: but a system to be tolerably perfect, should apply generally. Now, if we were to extend these designations to external inflammations, we should often find ourselves involved in difficulties which, in their consequences, amount to more than technical errors; for example, in classing erysipelas, can it be considered as an inflammation of the sthenic type? Is it not generally the reverse?—Can we class it as asthenic?—Would not this lead to a very erroneous consideration of the purer kinds of erysipelas phlegmonodes?

"The objection to this mode by no means rests on the support of this example only, but many others might be adduced. Now, by taking the disposition to limit or to spread, as the leading mark of distinction, we do not imply the necessity of *treating* them sthenically or asthenically; we merely recognise a prominent character, which ought to have a material influence over our conduct, and in the case of external inflammation I should strongly contend for the utility of doing so.

"The objections to employing the terms *phlegmonous* and *erysipelatous* are not less weighty. As phlegmon and erysipelas, are names now given to definite forms of disease, we naturally connect the idea communicated by the adjective phlegmonous with the disease which we entitle phlegmon, and so of erysipelatous. Now, if we were to include all the inflammations, whose disposition it is to be limited, under the former designation, we should ally with phlegmon those which are exceedingly dissimilar in nature, as for instance, mumps and carbuncle; and in the same way, if we called all those inflammations erysipelatous, whose disposition it is to spread, we should confound with erysipelas a variety of affections of the skin, of the vascular system, *&c.* we should have even paronychia gravis, whose seat is essentially *beneath* the skin, while erysipelas is more particularly resident in it. These and other examples are, I conceive, sufficiently strong to prove that such terms as these are liable to the very weighty objection of confounding together diseases essentially different in nature, while, by the mode now suggested, this is, as far as I can see, entirely avoided.

"*Secondly.* The degree of connexion of the organ with the vital functions of the animal, is another cause which exerts a predominant influence over the character of the inflammation; acts invariably, and *cæteris paribus*, in the same degree; the constitutional sympathy being in proportion to the danger, the difficulty of resisting that danger, and of repairing the mischief done. On this principle I would form the Orders.

"*Thirdly.* There is a circumstance in the history of inflammations which has hardly received a *due* share of attention, but it is both sufficiently remarkable, and very important; namely, the original disposition to terminate in one mode rather than another: thus, in boil and whitlow, it is to suppurate; in carbuncle to slough; and in mumps to resolve; and this disposition is so strong, that it is very difficult to procure any other termination. It may happen, however, that there shall be more than one mode in which it is disposed to terminate; as in either resolution or suppuration in sphacelus or ulceration, and so on. On this principle I have grounded the establishment of the Genera: whether it will fulfil the expectations of a strict classification is more than I can pretend to answer for; but of the practical importance of the principle I feel little doubt, or of its not having hitherto been carried to the extent it will admit." 17.

We must refer to the "Nosological Table of Inflammations," which is added to the work for the classification of the orders, genera, and species, admitted in the proposed arrangement.

The difficulties with which an author has to contend, who enters upon the task of forming a classification of so proteiform a state as inflammation, are so manifold, that we should be inclined to view, with peculiar indulgence, a less successful "attempt" than that which is submitted to the profession by Mr. James. The division of inflammation into two classes, founded upon its disposition to limit, or to spread, and the principles which guide our author in the formation of the orders, appear to us an improvement upon the previous arrangements of this subject. We cannot, however, but disapprove of the arbitrary separation of diseases of the *same name* and *situation*, merely because they occasionally differ in their degrees of violence. It is not likely that such distinctions "will practically be attended with any inconvenience," inasmuch as the practitioner is rarely influenced in his treatment, by the systematic arrangement of a disease; but if generally applied—if the several varieties of the same disease are to be thrown into different genera and classes, that perspicuity which should form the principal feature in every system of nosological arrangement, would be utterly destroyed. It is true that Mr. James does not carry the innovation of which we complain to any extent; as a precedent, however, it is dangerous, and to us the grounds appear insufficient, upon which he attempts to prove the propriety of deviating from every other classification of the subject by the separation of the different kinds of PARONCHIA and FURUNCULUS.

Upon the whole, we are of opinion, that if the arrangement of Mr. James, be considered with reference to those we previously possessed, the errors of which he has pointed out, its value, in our present state of knowledge at least, will be acknowledged and duly appreciated by his professional brethren. We do not venture to determine that it is unexceptionable; that it is comparatively so, is highly creditable to the talent of our author, when we consider the abilities of those who have devoted their attention to the attainment of the same object.

In Section I. chap. ii, a very brief sketch of the local symptoms of inflammation is given. The author is conscious that the account of the phenomena of inflammation, is very incomplete. We learn from the preface, however, that it is not his intention "to encumber his work with details, which are already before the public, by the labours of far abler hands."

In the second section of the same chapter "the supposed final causes of inflammation" are considered.

"That the vessels" observes our author, "are materially, and, indeed, principally, implicated in producing it, must be granted; and also, that we are not very likely to arrive at any very precise notions of their state and functions in inflammation, unless we are acquainted with them in health, which is so far from being the case, that there is, perhaps, no point in physiology more involved in obscurity. This being so, doubts may arise whether it would not be the most prudent plan to avoid the discussion altogether; and this opinion will receive further strength from the mortifying fact, that although men of the most exalted abilities and enduring patience have been engaged in the enquiry, it can hardly be considered to have led to any useful result." 23.

Under these circumstances Mr. James would "feel inclined to sit down without further investigation," did it not appear to him, that the doctrines which are now with much ingenuity maintained, and which are founded upon much research, seem to lead in their *practical application* to inferences, which are at variance with what he conceives to be the right treatment of the majority of inflammations, for he remarks,

"If it is granted that it depends upon debility of vessels, by consequence it would follow, that it should be our endeavour to counteract this debility."

The author is aware that neither Dr. Philip nor Dr. Hastings departs from the well-known principles of treatment in inflammation, which practice has established, but he fears that their doctrines will induce *others* to do so. Now we really perceive no reasonable cause for alarm upon this point; it requires but a very superficial consideration of the doctrines promulgated by the supporters of the debility of the capillary vessels, to be convinced of the impropriety of having recourse to a mode of treatment "at variance with that generally supposed to be required in the majority of inflammations."

In opposition to the authors whose names we have just mentioned, Mr. James is inclined to consider that the phenomena of inflammation are the result of *increased action.* He then takes a cursory view of those facts which seem to militate against the doctrine of debility.

It may be inferred, he thinks, from many circumstances, that the heart is not *essential* to the circulation; at least for a time it may be carried on without, though imperfectly; and it may be presumed that it does not *alone* support it in the human animal, under ordinary circumstances.

"Since, then, some other power is to be found to do this, the question is, where does that reside? It is maintained that the arteries contract to propel the blood; but if so, either they must contract together, or at different times; if together, they must either do so at the *same* time as the heart, or *alternately* with it; if at the same time, this would prevent the blood from flowing into them at all; if alternately, that would maintain the pulsatory action in the veins, whereas this is of rare occurrence. There is another mode in which they may be supposed to contract, namely, in succession, like an intestine; but as far as I have ever been able to find, both the dilatation by the impulse of the heart, and the contraction by the *consequent* exertion of the *elasticity* of the artery, are in immediate succession, and occur at the same moment, at any distance from the centre, which is at variance with this supposition; and as the elasticity of the arteries diminishes, so as to be almost imperceptible in the smaller branches, in them the pulsatory motion is gradually lost; whereas, if the contractile power operated, it would be continued, nay increased, as *their* contractile powers are stronger than in the larger vessels. Hence, as well as for many other reasons too long to specify here, I should be led to believe that the circulation is not maintained in any degree by the muscular contraction of the arteries; and I also believe that their power of contraction is given them for another purpose, quite sufficient in importance, namely, to *regulate* the quantity of blood conveyed through them to any part, or to the whole.

"It is unnecessary for me here to detail the reasons which induce the belief that a power of propulsion resides in the capillaries; but as the opinions which have been formed respecting the state of the vessels in inflammation have originated in some considerable degree from the notions which have been entertained respecting this power in the *arteries* also, it was necessary that I should express my dissent in this particular." 27.

It is observed that the theory of debility and diminished circulation in the vessels belonging to inflamed parts, as maintained by Vacca, Lubbock, and Allan, rests chiefly on the hypothesis, that the arteries propel blood ; and before it can be admitted, it is necessary that this power of propulsion in the arteries should be proved.

"It is not sufficient that an artery will contract—that it will contract under the application of a stimulus; it should be *proved that, under ordinary circumstances, it does propel the blood.*"

In answer to the experiments stated by Dr. W. Philip and Dr. Hastings, it is once more contended by our author, that microscopical observations are liable to mistrust, and that analogies between the higher and lower orders of animals, the chief subject of their experiments, cannot be deemed conclusive. It is granted that *dilatation* of vessels is a necessary part of the process of inflammation, but—

"I do contend," says Mr. James, "that *this dilatation is not essentially connected with debility:* it might as well be argued that the uterus dilates from debility to contain the fœtus, or the rectum to allow passage to the fæces. No doubt, in both these cases the cavities permit themselves to be distended; but having, by a temporary suspension of their action, or from the impression of a predominant force, suffered an alteration of their calibre, they immediately resume their usual degree of action on their contents, or probably act on them with more power than before. This may be equally the case with vessels enlarged from inflammation, or in another less exceptionable instance, *i. e.* when the branches of a tied trunk enlarge.

"So obviously incapable is the doctrine of debility of explaining the opposite states of inflammation, *i. e.* common or acute, and passive or weak, that its supporters have recourse to the *supposition* of the larger arteries possessing in the former an increased degree of action, but not in the latter; but that the capillaries are in a state of debility in both. Now, of course, if the propelling powers of the arteries are not proved, this must be doubted; and if disproved, fall to the ground." 32.

We must remark, in answer to the latter part of the passage just noticed, that the increased action of the larger arteries during inflammation is *not* a matter of supposition, nor does it rest upon the doubtful authority of microscopical observation; the increased pulsation of the large arteries may be seen without difficulty, when inflammation is seated externally, and is, we presume, a sufficient proof of their increased action.*

After having premised those arguments which oppose the doctrine of debility, the author proceeds to state *facts*, most of which, however, have been enforced by other writers upon this subject, which appear to him to support the contrary opinion. When we take into consideration the result of the various experiments, and the nature of the conflicting hypotheses, that have been instituted for the purpose of determining the state of the blood-vessels in inflammation, we are inclined to consider, in opposition to our author, that the doctrine of the debility of the capillaries *taken in all its parts*, is less liable to objections, than that which supports the idea of their increased action. Our limits prevent us from entering fully into the subject; we cannot, however, refrain from observing that Mr. James, who admits the *dilatation* of the vessels of an inflamed part; who admits also that they contain an *increased quantity of blood*, but who nevertheless believes that their action is *increased*, has not satisfactorily to us, at least, replied to the forcible argument of Dr. W. Philip, who observes, "When we speak of a morbidly increased action of vessels, do we allude to the state of their muscular coat? As this possesses transverse fibres, the effect of unusual contraction must be an unusual diminution of their area. Do we mean by morbidly increased action an increase of elasticity? the consequence of this can only be a greater tendency, in the vessel to preserve its mean area."

Let us not, however, be ashamed to confess, that upon this subject our knowledge is at present extremely limited; no theory has yet been promulgated which has not its vulnerable points, and until we arrive at a more perfect acquaintance with the *functions* of the *nervous* system—until we have the means of ascertaining the precise influence excited by the nervous over the vascular system, we must not hope to gain an accurate knowledge of the *nature* of inflammation. To determine by abstract experiments or otherwise, the state of the sanguiferous system, is doing little or nothing towards an elucidation of this interesting but intricate subject.

The 1st Section of Chapter 3, "On Sympathy," does not claim particular notice; it consists chiefly of a brief sketch of facts, which are as well known as they are indisputable. Sympathy is considered only as connected with disease. Mr. Hunter also treated of it in this point of view alone. He treated of universal and partial sympathy; of the continu-

* In this opinion, the Editor does not coincide with the Reviewer.

ous, contiguous, and remote; of similar and dissimilar. Mr. James conceives it would be more simple and satifactory to consider it under three heads:—1st. As "Sympathy of immediate Connexion." 2d. "General Sympathy." 3d. "Sympathy of Function." The influence of sympathy in producing the processes of inflammation, such as the effusion of organizable lymph, and the conversion of this into animal structure is remarked. It is observed also that the most simple instance which presents itself of the connexion of inflammation with sympathy, is to be found in cases of injury. The phenomena which occur locally are termed inflammation: those which take place generally constitute sympathetic fever. When the sympathetic actions of the part and of the constitution do not exceed a due degree, they are not only not injurious, but useful and necessary; but supposing they do exceed that degree which is so, what will be the consequence?—

"That much more blood will be brought to the part than it can dispose of, and with an undue degree of force, both which circumstances disturb the action of the minute vessels, and they cannot perform their task. But the efforts of the constitution are in proportion to the necessity for repairing the mischief and the difficulty in doing so; the struggle, therefore, on the part of the system, increases, as the mischief itself increases, from the very efforts made to remedy it, and disorganization, as it is termed, ensues, *i. e.* the vessels are so overstrained by the influx of blood, so disabled by over-exertion, compressed by surrounding effusion, or, perhaps, actually ruptured, that they are disqualified for their proper offices while fresh efforts are still made to obtain the object. Although, however, this cannot be directly accomplished, the vessels are, nevertheless, gifted with the power of effecting this indirectly in a great number of instances, by thé formation of granulations to which the secretion of pus is accessary; if, however, the repair has been obstructed by such over-action as now stated, that pus will be of an unhealthy nature; and if this end cannot be attained, the last resource of nature to terminate the struggle between the part and constitution, is, to remove it either by ulceration or mortification." 43.

That the degree and kind of actions sympathetically excited are much influenced by the nature of the injury, state of the constitution, and nature and state of the part, does not admit of doubt. Our author therefore touches briefly upon this part of the subject, and proceeds to notice the consequences which arise from continuity of surface, and contiguity of situation of inflamed parts. He conceives it unnecessary to dwell upon the nature of "Sympathy of Function, or Remote Sympathy." "General Sympathy," he observes, results from an affection of those systems which, by the extent and importance of their sympathies, are affected by every injury, and influence every function and process.

Sect. II. "*On the Accordance of the General and Local Affection,*" contains some interesting observations (for which we must refer our readers to the work itself) upon the probability that the affection of the system generally accords with that of the part in *kind*, if not in *degree*. Mr. Hunter was of a different opinion.

Dr. Thompson, in his "Lectures on Inflammation," p. 90, appositely observes, "No sooner do any of the subordinate parts of the animal economy receive an injury, or become affected with disease, than changes are induced in the general system, corresponding in some degree to the natural seat and extent of the local affection."

Chap. IV. "*On the State of the Digestive Organs,*" as connected with inflammation. Mr. James considers it superfluous to enter into details upon this highly important subject: he refers his reader, with laudable deference, to the works of Mr. Abernethy, observing, however, that—

"An impure state of the blood exists, perhaps, more frequently than we are aware of; but, as it is invariably connected with disorder of the digestive organs, the effects which partly arise from both causes, are often exclusively attributed to one. But when I see persons in whom every scratch festers into a sore, as in scurvy or scrofula;—when I observe that the atmosphere alone will change the disposition of every action;—that poisons introduced, and acting upon the circulating medium, will induce the most powerful effects upon the whole system; I must profess myself to be a humoralist in a considerable degree, (although quite ready to recognise the direct, as well as the indirect, influence of the digestive organs and nervous system on disease,) a doctrine which Mr. Abernethy has himself most perspicuously enforced, with reference to many; for, although he prefers the general explanation of the phenomena of disease by sympathy, yet he does by no means exclude the influence of a depraved state of the blood.

"If experimental research often instructs, I believe also it frequently deceives us, because we too hastily form deductions from what *we* deem *conclusive* experiments. Now, the opposition to the humoral pathology has chiefly been grounded on the fact, that we cannot discover such an alteration in the circulating fluids as would seem to us to justify the supposition that it exists. When, however, we see the efforts and the expedition with which the animal economy rids itself of *some* foreign matters, which we can recognise by their specific effects, as ipecacuanha or jalap introduced into the veins, or by their sensible qualities, as mercury, nitre, asparagus, turpentine, garlic, &c. introduced any how, we must be strongly prejudiced, not to believe that those matters have been contained in the blood—even although in the blood we cannot perceive them. But do not the vital powers quell and suspend the chemical properties of most substances?—Do they not convert the most dissimilar matters into an apparently similar substance?—May they not prevent these, and others, from manifesting their specific qualities, which, nevertheless, though modified, are not completely subdued, and may still excite great disturbance in the constitution?—And if so, may not these powers, with reason, be supposed to prevent the *sensible* alterations in the qualities of the blood, in such diseases as scurvy, scrofula, gout, lues, mercurial disease?—And if we are compelled to admit such alterations in these, may we not be called upon to allow that it possibly exists in a greater or less degree in many others? The abuse of the humoral pathology has led to infinite mischief; but if we are content to recognise the truth of the doctrine, as far as facts seem to establish it, without, however, grounding any theory or practice on that which we only imperfectly understand, no harm can arise from our belief. We can never err while we consider experience paramount." 59.

At page 62 the partiality of our author for the doctrines of the humoral pathology, is carried still further, for he says,

"If it be demanded, upon what system the air chiefly acts? I should again say, on the blood; for, if we take a man in previous good health, and place him in an hospital, for an injury not very severe, at a time when the cases there are doing badly, and find that, without any cause which can particularly affect his alimentary or nervous system, a bad kind of inflammation is set up, is it an unreasonable conjecture that the blood which, in the lungs, is particularly exposed to the contact of this air, should be thereby rendered impure? If we find that he does much worse when lying on the floor of the ward than if raised two or three feet above it, knowing that it is more deteriorated at the bottom than any where else, will not this opinion receive strength? And if, on sending him into a pure air, he does well, will not this add confirmation? It must be understood, however, that, although blood rendered impure by this, or any, cause, is likely to afford but a bad materiel for the processes going on in an inflamed part, yet that its operation on the nervous system, generally, and through this on the alimentary, is, perhaps, equally, if not more, injurious." 62.

The remaining pages of this chapter are occupied by a very brief consideration of "the state of the nervous system." The question as to the good or ill effects of weakness or strength, with reference to inflammation, is also touched upon.

"If strength is to be considered advantageous, inasmuch as it is the result of health; weakness is only to be feared when it arises from unsoundness of constitution. We need not be afraid of strength; because we have it in our power to reduce it. We need not seek to obviate weakness when it is merely the natural result of disease. If, for instance, we are to perform an operation rendered necessary by injury, although the patient may be rendered weak by the previous processes, we do not on that account think it desirable to give him tonics or cordials to prepare him for it. It seems to me to be of consequence to establish the point, that when weakness exists simply, without disorder, it will not lead to any mischievous consequences; and that strength, in as far as it is the offspring of sound health, is advantageous." 68.

We shall not dwell upon *Chap.* V. "*On the Purposes and Uses of the different Modes of Inflammation.*"

Mr. James is contented with giving a very short statement of the objects which Nature has in view, in the processes of inflammation. Dr. Thompson's, and Mr. Wilson's "lectures" are referred to as excellent epitomes of the knowledge we owe to Mr. Hunter, upon this subject, and of that which has since been added by the labours of subsequent investigators and themselves.

Chap. VI. "*On Mortification.*" The principal objects of the author in this chapter, are to point out the advantages which would result in a practical point of view, from a more particular arrangement of the different kinds of mortification than has hitherto been adopted, and to shew that this subject has hitherto been considered in too general a manner. Mr. James, we find, is not singular in his opinions upon this point. Dr. Thompson remarks, that "the various modes in which parts die, the diversity of causes from which their death proceeds, and the dissimilar or even opposite modes of treatment, which those affections in different stages and in different circumstances require, render a careful investigation, and an accurate arrangement of the subject, most important objects in the practice of surgery." The opinions of a recent French author, (M. Hebuardi,) in the "*Dict. des Sciences Med.*" *art. Gangrene,* are also in unison with those of Dr. Thompson and Mr. James upon this subject. The definition of the term mortification does not differ substantially from that of other writers.

"Mortification appears to be a peculiar alteration induced in the body, in which the fluids are coagulated, more or less perfectly, the solids changed in their texture, and the functions of the nerves and blood-vessels completely abolished; in short, a part so circumstanced is dead: but this is not simple death, from which the body, or a part, may, under some circumstances, be recovered; but it is an irreparable destruction of the organization and powers.

"Gangrene most commonly precedes this state, and, indeed, it may be doubted whether it does not in every case. But gangrene is, in point of fact, one mode of inflammation, and many authors have denied that inflammation is, of necessity, a precursor of mortification. One only, as far as I know, has advanced the opinion that it is, and with him I should agree in believing that it is preceded in general, and invariably *accompanied* with some degree of it.

"But whether mortification be a consequence of inflammation or not, it may, perhaps with reason, be considered as standing in the same relation to inflammation as adhesion, suppuration, or ulceration: they may all be preceded by a high degree, or it may be scarcely sensible. It is not improbable that they, as well as it, *may*, in some cases, be original actions, and that parts may adhere, suppurate, or ulcerate, without increased heat, swelling, or turgescence of vessels, without those symptoms which we denominate inflammation." 84.

We do not imagine that actual inflammation always precedes mortification; a part before it mortifies is in certain instances affected with pain only, and with no degree of preternatural redness. In the great majority of cases some degree of inflammation does *accompany* mortification; we have seen cases however, and we refer more particularly to two, both of which ended fatally where the disease was situated in the inferior extremities of men advanced in life, in which there were no accompanying appearances or symptoms which could fairly be said to constitute the inflammatory state. After enumerating the different kinds of mortification, which have been recognized, and for which particular modes of treatment have been separately specified, Mr. James observes—

"Lastly, we have mortification treated of as the result of inflammation, and here I think there is still a failure in our present systems, which, whether we look abroad or at home, practically divide it into two or three species.

"'For the sake of order,' says Dr. Thompson, 'I shall divide the observations I have to make on the cure of mortification; 1st, into those which relate to the treatment of acute mortification, and 2ndly, into those which relate to the treatment of chronic mortification, commencing without fever, or attended with fever of a typhoid type. It unfortunately happens,' he adds, 'that in mortification, as in many other diseases, there are cases of a mixed nature, in the conduct of which very decided modes of practice will be found to be as uncertain in their principles as they may be doubtful in their effects. P. 558.'" 89.

There are two modes of treating diseases, one by investigating their general principles, and speaking generally of the plan of cure; the other by considering each species individually and its appropriate treatment. The difficulty and importance of the former is duly appreciated by our author. He apprehends, however, that with reference to the present subject, the first mode has been adopted, almost to the exclusion of the latter.

"One author will include all he has to say under the head of acute or chronic gangrene, and their treatment; another, under the head of remedies, such as blood-letting, cold, heat, or the antiphlogistic plan, generally, and so on. I would not be understood to object to this proceeding, but to advance the opinion, that the work is left imperfect, unless, after the general scheme has been made out, the particular parts are distinguished. If, therefore, I were to attempt to write on this subject, it would be with the design of treating individually of mortification as produced by the application of certain causes, or as the result of certain inflammations; and in the present sketch it will be my object to do so as far as I well can." P. 93.

The propriety of dividing the fever which attends gangrene or mortification into three, or, at most, four kinds, is, in the opinion of Mr. James, very questionable. The difficulty of disturbing ideas once established is acknowledged, and it is presumed, that to multiply fevers by the number of different inflammations would probably never be tolerated.

"But let us get rid of the term fever, and state the fact in plain words, and it may perhaps be admitted that the constitutional sympathy varies as the local affection." 95.

Cases have fallen under our observation which would induce us to form a different opinion from that contained in the following passage—"when the state of gangrene is fully established, the character of the constitutional affection will be nearly the same, however different the cause may *be*," *(have been.)* We have seen well marked and important differences of constitutional affection, notwithstanding gangrene was fully established. The fact may be, as stated by Mr. James; in most instances, exceptions from it, however, are, we apprehend, not uncommon.

The chapter concludes with a brief enumeration of the causes which are capable of influencing or accelerating the progress of mortification, many of which may be avoided by attention or counteracted by proper means.

We now arrive at "*Part Second*," "*On the different Kinds of Inflammation*," in which are offered "a few brief observations on the general principles which may be applied to the various leading divisions of inflammatory diseases, and on the particular nature of some of the individual species. They may first be divided into those which are produced by external causes; 2dly, those which arise from a disordered state of the constitution itself." It is almost unnecessary to observe, that the arrangement proposed by the author, is adopted in his consideration of the different kinds of inflammation.

We have been so anxious to impart to our readers an ample knowledge of the first part of the work, which contains an elucidation of the principal objects of the author, that we must necessarily be brief in our notice of the second, in which many highly interesting subjects are touched upon with a brevity, that might induce a reader to accuse the author of having formed a very inadequate idea of the importance of the subjects on which he treats, if it were not remembered that—

"It is not pretended to give any thing in the shape of a full or a detailed account of any one kind of inflammation: to have done so would have swelled its bulk to a great and unnecessary extent; but in a few instances where there seemed to be *occasion* for enlarging more than is consistent with my *general* plan, I have done so." P. 142.

"Chap. I. *From Mechanical Injury.* Inflammation from mechanical injury is far from being one and the same in all cases; but, like other inflammations, differs in kind as well as degree, according to the nature of the cause, (the injury,) the part, and the constitution.

"Beyond doubt its invariable *object* is to repair the harm that may have been inflicted, although the endeavour often fails of success; it must, therefore, be considered as a *natural* process, but it does not always follow that it should be a *healthy* one. In this respect, however, it differs from spontaneous inflammation, for that of necessity implies disease, while this may occur in perfect health. It differs also in another important respect; namely, that it always has a disposition to a speedy termination; *i. e.* adhesion, suppuration, or slough; it also has universally a disposition to recovery. It is no objection to this statement that its termination is often prolonged by the presence of foreign matters, or its object frustrated by excessive violence.

"When the constitution is sound, and the injury not excessively severe, the inflammation is of the best kind, and generally succeeds in re-establishing the integrity of the part by the effusion of lymph or the formation of granulations. There is probably no inflammation in which there is absolutely no attempt to effuse organizable lymph; but in these, that process is invariably present to a great degree.

It is remarked that little or no sympathetic fever results from the infliction of mechanical injury, if the process of adhesion takes place uninterruptedly. The differences which arise in the inflammatory process from the nature of the organ concerned, are noticed. It is considered remarkable that tendons or ligaments, although incapable of bearing severe inflammation from external injury *with wound*, will do so in gout, rheumatism, or contusion. A few correct but brief observations follow upon the mechanical injuries of bones, and the different effects resulting from incisions, contusions, or lacerations. The principles of treatment laid down, although concise, are well worth attention.

In conformity with the plan of his Essay, Mr. James passes rapidly over the subject of "*Inflammation from Excess or Diminution of Temperature*," "*Inflammation from Poisons*," "*Inflammation of Vital Organs*," "*The Causes which produce Inflammation in Vital Organs*," "*Inflammation of Joints*," &c. &c. He conceives that the general principles of inflammation which it has been his endeavour to support, will find some confirmation in the phenomena exhibited in "*Inflammation of the Eye*," upon which subject he remarks that—

"In the first place, it is perfectly well known that when ulceration, abscess, or sphacelus, are spreading in the cornea, still more in the globe, the constitutional sympathy is far greater than when the mischief is arrested by the effusion of organizable lymph, and that if this can be procured by the use of arg. nitrat. remarkable relief is obtained with respect to the general symptoms, while on the other hand those remedies which will correct, subdue, or change, the state of the system, will influence the progress of the disease in a manner no less remarkable; and as these are processes which we can actually observe and watch, as much as if they were experiments contrived in the most delicate manner, they possess particular value. In the second place, this sympathy is much less when the conjunctiva, or integument, of the eye is alone concerned, than when the more essential parts are so, and there is risk of disorganization. And, in the third place, it may be noticed that each kind of inflammation of the eye has its own peculiar disposition to terminate in one mode or another:—in some, this is effusion of puriform mucus; in others, in vesicle and ulceration; in others, in adhesion or abscess; in others, in sphacelus; and in others again, to persist merely as inflammation." 163.

The subject of "*Inflammation of the Testis*," is dismissed with a few very brief observations; we notice it merely for the purpose of expressing our regret that a very efficient method of drawing blood from the part affected, by opening the enlarged scrotal veins with a lancet, is not more frequently had recourse to. It is not mentioned by our author, and we believe, indeed, that the practice is seldom adopted. In several cases of inflamed testicles we have drawn blood in this manner with much advantage. A greater quantity may, in some cases, be thus taken, with less trouble to the patient and practitioner than by the application of many leeches, independent of which, it is advantageous in an economical point of view, particularly in hospital practice. We should observe, that it occasionally happens, even when the part is much swoln, that there are no veins sufficiently apparent to allow of the abstraction of blood in the way we have just mentioned. We transcribe the following judicious observations upon "Inflammation affecting common or external parts."

"It is one principal object of this essay to ground the plan of treatment on the peculiar disposition of the inflammation we have to manage, and one of the most important features in this is, its tendency to one mode of termination rather than another.

"'A criterion,' says Dr. Kirkland (vol. i. p. 317), 'ought to have been fixed, when to attempt discussion, and when to let it alone.' I believe no better can be found than the natural disposition of the inflammation itself; for, if that is decidedly to suppuration, no attempt, probably, to resolve it will be successful, or, if so, advantageous, with a very few exceptions; for such a disposition is generally calculated to relieve the constitution.

"When the disposition admits either of resolution or suppuration, then of course the former is to be preferred, but the means of accomplishing this object are by no means the same in all cases; thus, where there is but little tendency to suppuration, we may employ warm applications without reserve; whereas, if there is much, they must often be avoided; while, on the other hand, they are particularly proper where there is *no* disposition to resolve. There is an avowed difference of opinion with respect to the superiority of warm or cold applications, and some surgeons are almost exclusively addicted to the use of the one or the other; but, where there is not such a prejudice existing, it has been confessed that the preference of the one or the other is very often founded rather on empirical than on rational motives. If the principles above stated are true, they may contribute to guide our judgment in this important matter." P. 170.

Some excellent practical observations are made upon the subject of "*Carbunculous Inflammation.*" The peculiar characteristic of inflammations of this genus, is their strong and almost irresistible disposition to terminate in the sloughing process more or less; and in the pus produced, being of the worst description and most irritating quality. They occur from the immediate effects of some local injurious cause; in most instances, in persons who have lived freely, grossly, and irregularly, and whose digestive organs are much disordered and loaded.

"The pyrexia, or degree of vascular reaction, will greatly depend upon the remaining powers of the constitution, and as the subjects are often people of originally strong stamina, it is often very considerable; but there is an invariable and strong tendency in all cases to sinking of the nervous powers, which, when sloughs are formed, often takes place very suddenly, and to a great degree. A hurried and anxious, and depressed state of the nervous system are manifested by the tremors, the tendency to low delirium, and the hurried and anxious countenance. The disordered state of the digestive organs is evinced by the furred and bilious tongue, the nausea, head-ach, and foul discharges.

"The object of medical treatment is to unload the bowels, which generally contain a large quantity of collected fæces, and at the same time procure secretions from the liver; a dose of calomel, followed by a mixture containing the sulphate of magnesia or tartrate of potash, senna in infusion and tincture, and jalap, with a little tartrite of antimony, if the nausea does not forbid it, perhaps afford the best mode of acting upon the bowels with these views; and, indeed, if there is any reason to believe that the stomach is loaded, an emetic is very useful. In the subsequent stages, blue pill, followed by rhubarb and senna, and tartrate of potash, form the best laxative, as I believe. The employment of purgative medicines suited to the case is by no means a light matter; in many instances it forms by far the most important part of the treatment, and if, by injudicious conduct, we leave accumulated fæces in the bowels, or fail to excite the secretions from the liver, or if we irritate them too much, and more particularly, if we bring on purging of watery stools, we shall do much harm where we might do much good. The compound extract of colocynth with calomel is often a good medicine in the beginning." P. 181.

In such cases, the patient must be supported according to his want of power; if we can keep him up till the sloughs form and separate, he will do well.

"*According, therefore, to the necessity*, it will be our duty to supply him with such articles of drink or food as may to us appear most advantageous, and in the advanced stages it often becomes necessary to push this plan pretty far; and indeed in all instances it is important to remember, that as these inflammations *are of the limited kind*, there will be less danger in exciting the vascular system than if they were otherwise.

"But it is of still more consequence to employ suitable local treatment, for upon this the life or death of the patient will generally depend. It is rarely possible to prevent these abscesses from going into suppuration, and therefore it should be our object to promote the process by warm fomentations, and poultices, which in some cases it is serviceable to make with stimulating fluids; for this kind of inflammation is attended with great want of nervous energy in the part, and for this reason, evaporating lotions or poultices would be highly improper." 182.

It is not only the character of such inflammations to form slough, but pus of the worst description; healthy pus simply excites a disposition in the part, to discharge it as a foreign body; but unhealthy, produces excessive irritation, and acts like a poison lowering its powers. There is a strong exertion made, to wall in matter so injurious, and this, contrary to the more general tendency, extends to the surface, and hence it is longer confined, a passage being only effected after a long time, by ulceration or mortification of the skin, if the patient lives.

Passing over the brief remarks of our author, upon Furunculus, Carbuncle, and Abscesses in the neighbourhood of the rectum and perineum, we arrive at Class Second, the character of which, is a disposition to spread from the failure of the adhesive process, owing to a faulty state of the constitution. It is not asserted that the disposition to limit is entirely absent in this class of inflammations: "on the contrary, there appears to be a struggle in the part and the general system, arising from the endeavour to effect that, which they are either totally, or for a long time, incapable of performing." Here then, the difficulty of the task which Mr. James has undertaken is apparent, for he confesses that the inflammation, which at one time belongs to his second Class, may, subsequently change its character, and claim a place in the first. Upon inflammation of the absorbent veins and arteries, Mr. James offers nothing peculiar to himself, in the few remarks he has deemed it necessary to make. Our limits oblige us to refer to the work itself, for the brief sketches which are made upon many of the remaining species of inflammation, which are comprised in the proposed arrangement. Upon the subject of erysipelas, will be found many interesting observations. It is justly observed that there is much difference of opinion, both with respect to the kinds of inflammation which ought to be included under this title:—the nature of true erysipelas, and the proper plan of treatment. It is defined by Mr. James, to be

> "An inflammation of the skin in every case, communicated to the cellular membrane in every species but one, the E. Erraticum; with a disposition to terminate in a few days, probably not exceeding seven or eight, either in resolution, or in suppuration, or sloughing of the cellular membrane, very probably both; and in vesications of the surface, accompanied with considerable tumefaction, chiefly from the secretion of serous fluids; and ending abruptly in the surrounding skin." 237.

The term erysipelas gangrenosum, as applied to a particular species, is considered objectionable, inasmuch as "it is a termination to which all are liable in a greater or less degree, and, therefore, it is erroneous to bestow this title on one kind only." It is justly observed, also, that the terms bilious and phlegmonoid ought not to be applied, as they are at present, in contradistinction to each other, for, certainly, the bilious character of constitution is often found combined with the phlegmonoid form of local affection. Mr. James does not attempt to reconcile objections to terms, which he looks upon as insuperable, but observes,

"I have taken the liberty of so adapting those we have, as to avoid any very palpable error as I hope; and have divided the E. phlegmonodes into two kinds, the more purely inflammatory, to which I have added the epithet verum, and the bilious; and to the latter have appended a variety which to me appears sufficiently well marked in which the absorbents are inflamed, under the title of E. phlegmonodes biliosum complicated with inflammation of the absorbents. The E. œdematódes will constitute another sub-genus, of which one principal species will be the E. gangrenosum of authors, which is the bilious erysipelas occurring in shattered constitutions." P. 239.

Having made such alterations in the titles, it necessarily follows, that the descriptions offered "*mutato nomine*," do not exactly accord with those which have heretofore been given under them.

The *treatment of erysipelas* recommended, is highly judicious; it does not materially differ from that which is enforced by the best practical authorities of the day. A very perspicuous, yet concise, account of the "Erysipelas Phlegmonodes Biliosum," combined with inflamed absorbents, is given. We are indebted to Mr. Copland Hutchison for the only particular account of this variety of the disease, which has yet been published. Mr. James does not appear to be very sanguine in his expectations of success, from the free use of incisions, which Mr. C. Hutchison has recommended; he remarks that, those cases which he has treated in this way, have not derived all the advantage from it, which he had been led to expect; but, on the other hand, the tendency of the wounds to run into mortification, *was not so great* as might, by some, have been apprehended. The pain and irritation which almost invariably accompany cases of this nature, are to be relieved by the free use of opium, the effect of which may be greatly assisted, by applying cold cloths to the forehead and head, which, without preventing its sedative influence, obviates its injurious impression, and often converts a forced sleep into a calm repose. The remaining pages of the work are occupied by a brief, yet correct description of "*Paronychia Gravis*" of "Inflammations tending to Phagedœna," and those which tend to mortification.

From the attentive perusal we have bestowed upon Mr. James's Essay, we are inclined to form a favourable opinion of his talents; he possesses the merit of supporting his own doctrines with much ingenuity, and is entitled to our commendation, for having honestly forborne to commit a literary fraud, which, in the present day, is not very uncommon—that of dressing up the opinions of others in his own words. The practical part of this book, although brief, is valuable; he has evidently extensive opportunities of witnessing disease, and is not without the requisite ability and industry to enable him to profit by his experience. Should a more sytematic work on inflammations ever issue from the same pen, which is indirectly promised in the preface to the present Essay, we venture to predict, with much confidence, that it will not only reflect credit upon the author, but confer a benefit upon the profession in general.

In looking over other works, which have been fortunate enough to gain the Jacksonián prize, we have occasionally been struck with a glaring inadvertency in the authors, from which Mr. James is entirely exempt. We have seen cases detailed with the names of the patients and consulting surgeons at full length, although the latter, would in all probability, be one of those who would ultimately decide upon the comparative merits of the work. This is improper in every point of view; it discloses the author as effectually, as if the regulations of the College with respect to the candidate Essay were paid no attention to, and *may* even create a suspicion of an undue bias and favour on the part of those, whose decisions *we* are quite certain, are influenced only, by the strictest impartiality.

